# Transhiatal *vs* extended transthoracic resection in oesophageal carcinoma: patients' utilities and treatment preferences

**DOI:** 10.1038/sj.bjc.6600203

**Published:** 2002-03-18

**Authors:** A G E M de Boer, P F M Stalmeier, M A G Sprangers, J C J M de Haes, J W van Sandick, J B F Hulscher, J J B van Lanschot

**Affiliations:** Department of Medical Psychology, Academic Medical Center, Meibergdreef 15, 1105 AZ Amsterdam, The Netherlands; Radiotherapy/Nijmegen Institute Cognition and Information, Nijmegen, The Netherlands; The Joint Center for Radiation Oncology Arnhem-Nijmegen (RADIAN), University Center Nijmegen, Nijmegen, The Netherlands; Department of Surgery, Academic Medical Centre, Amsterdam, The Netherlands

**Keywords:** utilities, treatment preference, oesophagectomy

## Abstract

To assess patients' utilities for health state outcomes after transhiatal or transthoracic oesophagectomy for oesophageal cancer and to investigate the patients' treatment preferences for either procedure. The study group consisted of 48 patients who had undergone either transhiatal or transthoracic oesophagectomy. In an interview they were presented with eight possible health states following oesophagectomy. Visual Analogue Scale and standard gamble techniques were used to measure utilities. Treatment preference for either transhiatal or transthoracic oesophagectomy was assessed. Highest scores were found for the patients' own current health state (Visual Analogue Scale: 0.77; standard gamble: 0.97). Lowest scores were elicited for the health state ‘irresectable tumour’ (Visual Analogue Scale: 0.13; standard gamble: 0.34). The Visual Analogue Scale method produced lower estimates (*P*<0.001) than the standard gamble method for all health states. Most patient characteristics and clinical factors did not correlate with the utilities. Ninety-five per cent of patients who underwent a transthoracic procedure and 52% of patients who underwent a transhiatal resection would prefer the transthoracic treatment. No significant associations between any patient characteristics or clinical characteristics and treatment preference were found. Utilities after transhiatal or transthoracic oesophagectomy were robust because they generally did not vary by patient or clinical characteristics. Overall, most patients preferred the transthoracic procedure.

*British Journal of Cancer* (2002) **86**, 851–857. DOI: 10.1038/sj/bjc/6600203 www.bjcancer.com

© 2002 Cancer Research UK

## 

In Western countries, carcinoma of the oesophagus is a relatively uncommon tumour. It disseminates early and most patients present with advanced disease. When cure can be expected, surgical resection is the therapeutic modality of first choice (Akoh and McIntyre, 1992; Devesa *et al*, 1998). Curative oesophageal resection may be carried out by either a transhiatal or transthoracic technique (Tilanus *et al*, 1993). The first strategy aims to decrease early post-operative morbidity and mortality by limiting the extent of the operation. The other strategy aims at improving the cure rate by performing a more radical resection.

Regardless of the surgical approach, oesophageal resection is a major operation. It is associated with severe physical and emotional effects and it may thus seriously affect the patient's quality of life. Both types of operation are accompanied by an extended hospital stay with possible postoperative morbidity such as respiratory infections. Afterwards, patients may face a lengthy recovery period, severe side effects of the intervention and the threat of recurrent disease. Recognising this health burden, researchers are increasingly studying the quality of life of these patients (Gelfand and Finley, 1994; Blazeby *et al*, 2000).

To evaluate the cost-effectiveness of both surgical strategies, patients' utilities for the outcome health states of oesophageal cancer associated with either transhiatal or transthoracic surgery are needed (Gold *et al*, 1996; Weinstein *et al*, 1996). Utilities range from 0 (death) to 1 (perfect health) and patients are typically asked to assign a value to their own current health state or to other potential outcomes of treatment. To our knowledge, utilities for potential health state outcomes of oesophageal cancer have never been assessed.

Apart from the inclusion of patient utilities, the explicit choice of patients for a particular treatment should also be taken into account when deciding which treatment to choose. These choices are known as treatment preferences and typically involve informed patients (De Haes and Stiggelbout, 1996; Keulemans *et al*, 1998; Nieuwkerk *et al*, 1998; Awad *et al*, 2000). The treatment preference method is a realistic reflection of the actual decision situation. Therefore, this method is especially useful to support the decision analysis.

The objectives of this study were: (1) to assess patients' utilities for health state outcomes after oesophagectomy of patients who had undergone either transhiatal or transthoracic resection and to explore how patient characteristics and clinical factors influence these utilities; (2) to investigate the treatment preferences of the patients for either transhiatal oesophagectomy or transthoracic oesophagectomy in the treatment of oesophageal cancer and to explore how various patient characteristics and clinical factors influence these treatment preferences.

## MATERIALS AND METHODS

### Patients

Between January 1997 and March 1999, 93 consecutive patients with adenocarcinoma of the oesophagus or oesophagogastric junction were asked to participate in the present study. All patients took part in a randomised clinical trial comparing transhiatal oesophagectomy *vs* transthoracic oesophagectomy with two-fields lymphadenectomy (Van Sandick *et al*, 1998). The study was performed in two academic centres (Academic Medical Center/University of Amsterdam and University Hospital Dijkzigt-Rotterdam). In both centres, the study was approved by the institutional medical ethics committee. All patients were provided written informed consent at study entry.

Inclusion criteria were: (1) age >= 18 years; (2) invasive adenocarcinoma of the middle or distal oesophagus or oesophagogastric junction and (3) locally resectable disease without distant metastases on preoperative investigations. Exclusion criteria were: (1) a previous diagnosis of other malignant disease; (2) organ insufficiency as defined by ASA III or IV (American Society of Anaesthesiology) (Owens *et al*, 1978); (3) impossibility to construct a gastric tube and (4) previous chemotherapy, radiation therapy or immunotherapy. Oesophageal resection was performed by a transhiatal approach without thoracotomy or via a right-sided thoracotomy in combination with a laparotomy. Gastro-intestinal continuity was usually re-established by constructing a gastric tube. In all patients, the anastomosis was performed in the neck.

### Interview

Patients were interviewed at the outpatient's clinic when the patient was scheduled for a follow-up visit. Patients with irresectable disease at the time of operation and those with recurrent cancer were excluded from the interview, because of possible emotional burden. Interviews took place 3–12 months after the operation, to optimise the chance that patients were in a stable situation with near-maximal functional recovery. All interviews were performed by one of two trained interviewers between May 1997 and October 1998. On average, the interview lasted 58 (range 35–140) min.

### Health state descriptions

**Figure 1:**
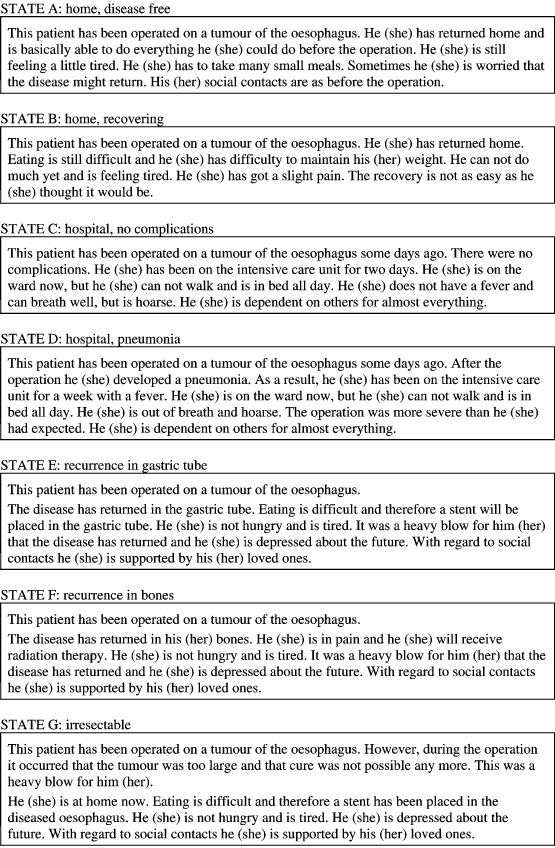
Possible outcome health states after oesophagectomy for oesophageal cancer.

Seven health state descriptions of possible outcomes after a transhiatal or transthoracic resection for oesophageal cancer were constructed (Figure 1

). The health state scenarios employed short phrases to convey information on specific areas of physical, emotional and social functioning. The areas included: (1) mobility; (2) disease free or recurrent disease; (3) pain; (4) hoarseness; (5) tiredness; (6) pneumonia (yes/no); (7) swallowing and number of meals; (8) psychological problems, and (9) social support. Using the patient's own gender, i.e. ‘he’ for men and ‘she’ for women, each description was read to the patient by the interviewer in random order and repeated on request. Also included in the interview was a description of the patient's own current health state. For this description, patients were asked to think about their physical, emotional and social health state of the past week. During the interview, patients were first asked to rank all health state descriptions including their own from 1 ‘best health state’ to 8 ‘worst health state’. Then, they were asked to assign utilities to the health state descriptions with either the visual analogue scale or standard gamble method and to indicate their treatment preference, as described below.

### Visual analogue scale (VAS)

A Visual Analogue Scale (VAS) was used as the initial method by which values were elicited for the patient's own current health state and the above mentioned health state descriptions. The VAS is a 100 mm horizontal line. The anchors were ‘worst imaginable health’ at zero and ‘perfect health’ at 100. Each subject was asked to sign a cross on the VAS for each health state. This method is easy to understand (Stiggelbout *et al*, 1996), but the scores produced are often not regarded as true utilities, because there is no risk involved.

### Standard gamble

The standard gamble is regarded as the gold standard for eliciting individual utilities because it is based on solid theoretical foundations and it involves risk (Gold *et al*, 1996). In the standard gamble procedure the patients are asked to choose between a certain-but-imperfect health state and a gamble between perfect health and death. In the gamble there is a probability, ‘p’, of living in perfect health and a probability, ‘1-p’, of death within a week. The value of ‘p’ at which the participant is indifferent between the gamble and a certain health state is the utility of that certain health state.

In this part of the interview, patients were introduced to a ‘chance board’ used to elicit standard gamble utilities. Probabilities for the gamble were presented in a ‘ping-pong’ fashion, beginning with a 100% chance of ‘perfect health’ and a 0% chance of ‘immediate death’, followed by 0% chance of ‘perfect health’ and a 100% chance of ‘immediate death’, followed by a 95% chance of ‘perfect health’ and a 5% chance of ‘immediate death’, and so on until the participant reported indifference between the gamble and the certain health state outcome.

### Treatment preference

In order to elicit treatment preference, the interviewer first repeated the information about both the transhiatal and the transthoracic procedures that was given to the patient as part of the informed consent procedure prior to the study inclusion. This information included: ‘It is unknown which of the two procedures is best. Theoretically, the advantage of the transhiatal method is that recovery after the operation might be quicker. The advantage of the transthoracic procedure might be that the tumour might be removed better as well more lymph nodes which might be affected by the tumour. Therefore the course of the disease might be more favourable. Disadvantage of the transthoracic procedure is that an extra wound is needed and that during the first part of the operation the function of the right lung will be temporarily eliminated.’

After reading this information, patients were asked to imagine a neighbour or friend with an oesophageal tumour who had to be operated upon, and to choose the preferred treatment for this other patient.

### Additional measures

All patients received a self-report questionnaire prior to the operation. The questionnaire included questions on marital status, having children, and education (primary school, high-school or college). During hospitalisation, clinical data were registered including (1) pneumonia, (2) other respiratory complications, (3) anastomotic leakage, (4) cardiac complications, (5) wound infection, (6) chylothorax, (7) ventilation time in days, (8) intensive care unit (ICU) stay in days, and (9) hospital stay in days.

### Statistical analysis

All data were checked for accuracy and analysed using the Statistical Package for the Social Sciences (SPSS-10.0). VAS scores did show a normal distribution and therefore parametrical tests were performed for VAS data. None of the standard gamble data followed a normal distribution as judged by graphical assessment and the Kolmogorov–Smirnov test for normality, nor was it possible to transform the data to fit normal distribution. Therefore, we used non-parametric tests for these data.

Differences in clinical data between the THE and TTE group were tested with the Mann–Whitney *U* statistic. For each health state, a Wilcoxon's matched pairs test was used for comparing mean utilities produced by the VAS and standard gamble methods. To determine whether the utilities of the individual health states were significantly different from each other, we used the repeated measures ANOVA and *t*-tests for the VAS utilities, and Friedman tests and Wilcoxon tests for the standard gamble utilities.

To determine whether patient characteristics were related to utilities, Spearman's rank correlations were calculated between utility scores and patient characteristics (gender, marital status, having children, education, age and months of follow-up when interviewed). To test for differences in utilities between patients with either transhiatal of transthoracic procedure and between patients with and without complications, one-way ANOVA was performed for VAS scores and Mann–Whitney *U*-test for standard gamble scores. The relationship between utilities and clinical data was further explored by correlating both VAS scores (Pearson correlations) and standard gamble scores (Spearman correlations) with length of ventilation, length of ICU stay and length of hospital stay.

To test for differences in treatment preferences between the two surgical groups, patient characteristics were tested with the χ^2^-test (gender, marital status, having children and education) or Spearman's rank correlations (age and months of follow-up when interviewed). Additionally, the effect of having experienced complications on subsequent treatment preference was evaluated by means of the χ^2^ statistic.

*P*-values <0.05 were considered statistically significant. Following recommendations of Cohen, correlations will be considered low if *r*<0.20, moderate if 0.20<=*r*<=0.50 or high if *r*>0.50 (Cohen, 1977). Only significant correlations are reported.

## RESULTS

### Patients

Of the 93 patients who consented to participate, four (4%) patients had deceased in the hospital and nine (10%) patients had died before the interview because of recurrent disease. Furthermore, 21 (23%) patients were still alive but were excluded because of recurrent disease. Of the remaining 59 patients, six (10%) ultimately refused participation because of emotional burden and three (5%) could not be reached. Therefore, 50 patients were interviewed (85%).

Of the interviewed patients, 26 (52%) patients underwent the THE procedure and 24 (48%) patients underwent the TTE procedure. Twenty (47%) of the excluded patients underwent the THE procedure and 23 (53%) the TTE procedure.

Data of two interviewed patients were excluded because of erratic data collection and because of cognitive problems with the standard gamble, resulting in 48 evaluable cases. Most patients were male (90%), married (86%), had children (91%), and had finished high-school (45%) or college (20%). The average age was 63 years (44–79 years) and average time of follow-up when interviewed was 7 months (3–12 months) after the operation. Clinical data of both THE and TTE groups are reported in Table 1

**Table 1:**
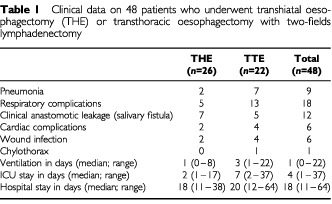
Clinical data on 48 patients who underwent transhiatal oesophagectomy (THE) or transthoracic oesophagectomy with two-fields lymphadenectomy

. Pneumonia and respiratory infections were significantly less frequent after transhiatal resection than after transthoracic resection (*P*=0.04 and 0.005, respectively).

### Utilities

The ranking and the VAS and standard gamble scores of the seven possible health states and the ‘own current health’ are given in Table 2

**Table 2:**
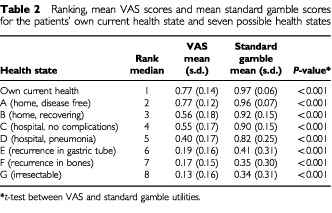
Ranking, mean VAS scores and mean standard gamble scores for the patients' own current health state and seven possible health states

. Highest ranking and utility scores were found for the patients' own health state. Regarding the possible health states, highest ranking and highest VAS and standard gamble scores were given for the disease free health state while lowest ranking and utilities were elicited for the health state involving an irresectable tumour.

The VAS method produced lower estimates than the standard gamble method for all health states (all *P*<0.001). A significant overall difference was found among the health state utilities for each assessment method (both ANOVA repeated measures and the Friedman test (*P*<0.001)). This would indicate that at least one health outcome state was distinct from one of the others in each elicitation method. Regarding the VAS scores, results of the *t*-tests showed most health states were distinct from each other. However, no difference was found between the own current health state and the disease free health state (*P*=0.82), between the health states ‘recovering at home’ and ‘hospitalisation without complications’ (*P*=0.67), and between the health states ‘recurrent disease in gastric tube’ and ‘recurrent disease in bones’ (*P*=0.15). For the standard gamble scores, results of the Wilcoxon test showed that again most health state valuations were different from each other. No differences were found between own current health state and ‘disease free health’ (*P*=0.11), between the health states ‘recovering at home’ and ‘hospitalisation without complications’ (*P*=0.22), and between the health states ‘recurrent disease in bones’ and ‘irresectable tumour’ (*P*=0.51).

### Utilities and patient characteristics

No significant correlations were found between marital status, education or months of follow-up on the one hand and utility scores for any health state on the other. Moderate correlations were found between female gender and standard gamble score for ‘recurrent disease in gastric tube’ (*r*=0.32), between having children and the VAS scores for ‘recurrent disease in bones’ (*r*=0.34) and for ‘irresectable tumour’ (*r*=0.33), and between higher age and the standard gamble score for ‘hospitalisation without complications’ (*r*=0.40).

### Utilities and clinical characteristics

As depicted in Table 3

**Table 3:**
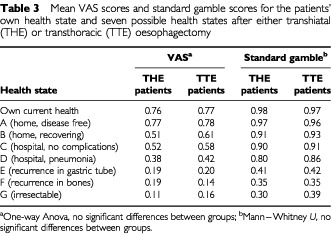
Mean VAS scores and standard gamble scores for the patients' own health state and seven possible health states after either transhiatal (THE) or transthoracic (TTE) oesophagectomy

, there were no statistically significant differences between patients who had undergone either the transhiatal or the transthoracic procedure in either VAS or standard gamble scores of any health states. In addition, the experience of post-operative complications had no significant effect on the utility scores for any of the seven health state scenarios. Length of ventilation, length of ICU stay and length of hospital stay were not significantly associated with either VAS or standard gamble utility scores on any health states.

### Treatment preference

In Table 4

**Table 4:**
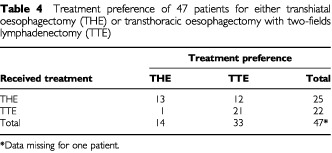
Treatment preference of 47 patients for either transhiatal oesophagectomy (THE) or transthoracic oesophagectomy with two-fields lymphadenectomy (TTE)

the treatment preferences are reported. The great majority of patients (21 out of 22) who underwent a transthoracic procedure would recommend the same procedure for another patient. However, half of the patients (12 out of 25) who underwent a transhiatal resection would not recommend a transhiatal procedure but transthoracic surgery. No significant associations between any patient characteristics or clinical factors and treatment preference were found.

## DISCUSSION

The main objective of this study was to assess patients' utilities for different outcomes after oesophagectomy by using a clinically relevant study population. We found that patients were able to order the presented health states as anticipated by the degree of described impairment in physical, emotional and social functioning and to provide VAS and standard gamble scores. The valuations they provided were of similar magnitude as utilities found in earlier research on cancer patients. Ness *et al* (1999) measured standard gamble scores for outcome states of colorectal cancer in a sample of 81 patients who had previously undergone resection of colorectal carcinoma. They found standard gamble scores of 0.84 for ‘own health’, 0.74 for the health state ‘disease free after resection’ and 0.25 for ‘metastatic/irresectable’. For these health states, we measured standard gamble utilities of 0.97, 0.96 and 0.34, respectively. In a study by Stiggelbout *et al* (1994), 30 disease free testicular cancer patients were interviewed with the standard gamble technique. They found a mean standard gamble score of 0.93 for the health state ‘disease free after orchidectomy’, which is similar to the 0.96 standard gamble utility we found for ‘disease free after oesophagectomy’. Our ‘own current health state’ VAS and standard gamble scores of 0.77 and 0.97 were also comparable with those of other chronic diseases: 0.68 and 0.91 for asthma (Blumenschein and Johannesson, 1998) and 0.81 and 0.92 for angina (Lalonde *et al*, 1999).

We consistently found significantly lower VAS scores as compared to standard gamble scores for the same health states. This is a well-known phenomenon (Froberg and Kane, 1989; Bass *et al*, 1994; De Haes and Stiggelbout, 1996) possibly caused by risk aversion (Froberg and Kane, 1989) or biased risk perception (De Haes and Stiggelbout, 1996). Risk aversion refers to the conservatism most people experience with respect to risk taking, while the effect of risk perception refers to an overestimation of low percentages. Both effects might lead to higher standard gamble utility values.

In this study, most health state valuations were found to be statistically distinct from each other. However, with both VAS and standard gamble techniques, no difference was found between own current health state and the disease free health state nor between the health states recovering at home and hospitalisation without complications. On the one hand, it was expected that the patients' own health state and a disease free health state would be valued similarly, because most patients were in a disease free health state after maximal rehabilitation at the time of the interview. On the other hand, people tend to value their own health state more positively than a similar possible one (Jansen *et al*, 2000). However, this last discrepancy did not occur in our study.

It was surprising that the utilities for ‘recovering at home’ and ‘in hospital after an oesophagectomy without complications’ were not distinct. We expected that patients are in a better health state at home than in hospital and that they would also prefer to be at home. It might be possible that the recovery at home after such a major operation is physically and mentally very demanding and is much more difficult than the patients had anticipated. It is of clinical interest that we did find a distinction between the two health states ‘hospitalisation without complications’ and ‘hospitalisation with pneumonia’. Significantly less patients suffer from pneumonia after transhiatal resection than after transthoracic resection (Van Sandick *et al*, 1998). Therefore, these different valuations might exert an effect in a cost-utility analysis comparing both treatments.

The utilities we found in our study were robust, because they were hardly influenced by patient or clinical characteristics such as age or respiratory complications. Several other studies have investigated the effect of patient or clinical factors on utilities. No evident effect of these factors on utility scores was found in these studies either. For example, in a study of 82 psoriasis patients, no patient characteristics and indicators of disease severity were predictive of utilities for the assessed health states (Zug *et al*, 1995). Standard gamble values of diabetic patients were found to be influenced by gender (Brown *et al*, 1999), but not by education or vision loss. In stroke patients (Hallan *et al*, 1999), only two of 11 parameters (marital status and age) showed a significant effect on SG scores. In a study of 51 HIV patients (Tsevat *et al*, 1999), a relation between disability and standard gamble scores was found, but 15 other variables had no effect on utility.

The fact that we barely found a relation between utilities and other factors could be caused by the fact that many characteristics did not show an even distribution of outcomes: most patients were male, older, married and had children. Furthermore, we examined a relatively small sample of patients and most patients did not have any severe complications during hospitalisation. However, the lack of effect of background factors on utility scores is so consistent in the literature, that it is probable that people base their valuations on the possible health state descriptions and are not influenced by background characteristics.

With regard to the treatment preference, half of the patients who underwent the transhiatal procedure would not choose their own treatment again, but the transthoracic treatment instead. However, almost all patients who underwent the transthoracic procedure would choose the extensive procedure again. This is a remarkable result, because earlier studies (Keulemans *et al*, 1998; Nieuwkerk *et al*, 1998) showed that people tend to choose the treatment they have received, even if this treatment was randomly allocated. In the present study, patients apparently want to optimise the chances that the malignant disease is definitely cured. They are prepared to undergo an even more demanding procedure to increase their chances in the long term. This preference for the transthoracic procedure might be biased by the informed consent information given to the patients. The information is based on the assumption that the transthoracic procedure might lead to favourable outcomes in the long term. However, this hypothesis is still being investigated.

One problem of the present study relates to the number of excluded patients which was over half the patients. Although the percentage of patients undergoing the transhiatal or transthoracic procedure was the same in the interviewed and excluded patients, the exclusion of patients with the worst postoperative course (in-hospital deaths and recurrence within the first year) might have biased the data. The health state utilities and treatment preferences might therefore not be generalised to all oesophagectomy patients, but only to the patients who survived the first year after the operation.

In conclusion, we have obtained specific estimates of utility values that patients would assign to outcomes after transhiatal or transthoracic oesophagectomy. We have also demonstrated that these robust utilities, either derived by VAS or by standard gamble techniques, generally do not vary significantly by patient and clinical characteristics. Finally, we have found that most patients would prefer the extended transthoracic procedure. These results should be incorporated into decision analysis and evaluation of cost-effectiveness of surgical procedures for oesophageal cancer.
